# Skin endothelial cell and microcirculation function study in recurred keloids patients after keloid surgery and radiotherapy

**DOI:** 10.1097/MD.0000000000031286

**Published:** 2022-10-28

**Authors:** Wenbo Li, Mengjie Shan, Yan Hao, Hao Liu, Youbin Wang, Jie Qiu

**Affiliations:** a Department of Radiation Oncology, Peking Union Medical College Hospital, Beijing, China; b Department of Plastic Surgery, Peking Union Medical College Hospital, Beijing, China; c Chinese Academy of Medical Sciences & Peking Union Medical College, Beijing, China.

**Keywords:** Keloid, microenvironment, radiotherapy, VEGF

## Abstract

Keloid is a type of benign tumor of the skin with abnormal proliferation of fibrous tissue. We sought to observe the changes in skin microcirculation and endothelial cell function around the recurred keloid and explore the skin microcirculation characters in recurred keloid patients.

**Methods:**

Six patients with recurred keloid were treated with keloid surgery and radiotherapy for the second time. Microcirculation of recurred keloids and their surrounding normal skin tissue was observed with laser Doppler flowmeter before operation. Expression of vascular endothelial growth factor (VEGF), CD31, and HIF-1α were identified by several assay.

**Results:**

The local blood flow of group RN was enhanced. The average strength of group N is 0.87. The average strength of group RN is 2.08. The expression of VEGF, CD31, and hypoxia inducible factor-1α (HIF-1α) protein in the keloid-recurred skin (RN) group was higher than the normal skin group via immunohistochemistry (IHC) and Western blotting analysis. The relative expression of VEGF and CD31 mRNA was significantly increased in RN group samples (*P* < .05).

**Conclusions:**

There are significant differences in the expression of VEGF, CD31, and HIF-1α in the recurred keloid skin after radiotherapy and normal skin. They may be used as potential biomarkers and targets for future research on keloid recurrence.

## 1. Introduction

Keloid is a benign tumor of the skin mainly characterized by abnormal proliferation of fibrous tissue.^[1–3]^ Keloid are often secondary to trauma, infection and post-operative, and are likely to occur in areas with high skin tension or repeated injuries, such as the parasternal, earlobe, upper arm, and back of the ear. It is less common in the palms, scrotum, penis and upper eyelids.^[4]^ Surgical resection of lesions assisted by local electron beam therapy (radiotherapy) is currently a relevant effective treatment method. Shen^[5]^ reported that 6 or 7MeV electronic beam were used to treat 834 keloids in 568 patients with an effective rate of 88.25%.

Electron beam therapy is the use of radiation to irradiate tissues to generate secondary electrons in tissue cells, which destroy the molecular structure of DNA through direct or indirect ionization, thereby inhibiting cell division and proliferation.^[6]^ The mechanism of radiotherapy to inhibit keloids may be various. Firstly, radiotherapy can inhibit the migration, proliferation and collagen synthesis and secretion of fibroblasts after surgery. Secondly, many studies have shown that inflammation is an important factor in the formation or recurrence of keloids. Radiation therapy can strongly inhibit the inflammatory response by weakening the function of immune cells and reducing the formation of dysfunctional blood vessels.^[7]^

The development process of keloids is divided into proliferative phase, regression phase and mature phase. Angiogenesis runs through each process, which can increase the number of new blood vessels and affect the inflammation and cell apoptosis in local tissues, thereby promoting the hyperplasia of scar tissue and the development of keloids. The abnormal growth of granulation tissue in horses and human keloids both show fibroblast apoptosis defects and microvascular dysfunction caused by luminal occlusion.^[8]^ Endothelial cell and microcirculation function after radiotherapy might be potential factor in determining the treatment results and recurrence rate. The effect of radiotherapy on endothelial cell function is still unclear. In the meantime, the influence of radiotherapy on skin microcirculation in the recovery process need to be studied.^[9,10]^ We aimed to observe the changes in skin microcirculation and endothelial cell function at the electronic beam treated skin site in post operation recurred keloid patient to explore their factors on keloid treatment.

## 2. Methods

### 2.1. Patients and sample collection

This study was approved by the Medical Ethics Committee of Peking Union Medical College Hospital (Medical Ethics Number:JS-2907). Six patients with recurred keloid were treated with recurred keloid surgical removing and radiotherapy for the second time from January 2019 to April 2020 (Table 1). Radiotherapy treated skin tissue (with obvious hyperpigmentation) and the surrounding normal skin tissue (without obvious hyperpigmentation) around the recurred keloids were obtained after operation. All the removed skin tissue were redundant tissue which had to be removed during operation. Operation consents and removed tissue donate consents were signed by all the patients.

**Table 1:** Characteristic of patients.

Patients^*^	Age of onset (yrs)	Gender
N1	21	Female
N2	19	Male
N3	26	Female
N4	21	Male
N5	34	Female
N6	44	Male
RN1	31	Female
RN2	32	Female
RN3	34	Male
RN4	27	Female
RN5	36	Male
RN6	23	Female

mVSS: The Modified Vancouver Scar Scale is used for the descriptive assessment of keloids, including melanin (M), height (H), vascularity (V) and pliability (P).

* K, N, and I samples were collected from a total of 5 patients.

### 2.2. Microcirculation analysis method

Microcirculation in radiated skin and surrounding normal skin was measured with a laser speckle contrast imaging (LSCI) system (PeriCam PSI System, Perimed, Jāfālla, Sweden). The measurements were carried before operation. The patients rested at room temperature (25°C) during measurement. The distance between the laser head and a patient’s skin was 20 cm. Measurements were carried out in each location for at least 5 seconds with 5 Hz laser frequency. Recorded images were analyzed using the software system (PimSoft 1.2.2.0, Perimed, Jāfālla, Sweden). The average value of blood perfusion in each site was calculated.

### 2.3. Hematoxylin-eosin (HE) stain

The skin tissues (removing the subcutaneous fat layer) were put in a pre-prepared fixative (10% formalin) to denature and coagulate the protein. After the fixation, put it into the embedding box and rinse with running water (remove the fixative in the tissue) for 30 minutes. Then, different concentrations of alcohol were used as a dehydrating agent to gradually remove the water in the tissue mass. The tissue was then transparent and embedded in wax. The embedded tissue block was cut into thin slices about 4 µm in thickness and then pasted on the glass slides. After dewaxing and dehydration, the slice was dyed for HE assay.

### 2.4. Immunohistochemistry (IHC) for VEGF, CD31, and HIF-1α

Paraffin sections were made using the tissue samples of the above samples. The paraffin sections were deparaffinized with water, sealed with hydrogen peroxide, then washed with double distilled water. VEGF, CD31, and HIF-1α were detected by using IHC after antigen retrieval. Anti-VEGF polyclonal antibody (Servicebio, Wuhan), anti-CD31 polyclonal antibody (Servicebio, Wuhan) and anti- HIF-1α polyclonal antibody (Servicebio, Wuhan) were applied. According to the instructions of the VECTASTAIN Elite ABC Kit (Vector Laboratories, Burlingame, CA), the specific detection steps were performed as follows. First, antigen-fixed paraffin sections were washed with phosphate-buffered saline (PBS) 2 to 3 times (5 min/times) and blocked with 10% goat serum (TransGen Biotech, Beijing, China) at 37°C for 20 minutes. Second, removed the serum by using filter paper, and added YAP or TAZ Rabbit polyclonal antibody (Abcam, Cambridge, UK) dropwise, then incubated overnight at 4°C. Third, the sections were washed with PBS 3 times (5 min/time) and incubated with goat anti-rabbit monoclonal antibody at 37°C for 1 hour. Fourth, color development was performed with diaminobenzidine. Each paraffin section was photographed. Anti-VEGF polyclonal antibody (Servicebio, Wuhan, 1:800), anti-CD31 polyclonal antibody (Servicebio, Wuhan, 1:800) and anti- HIF-1α polyclonal antibody (Servicebio, Wuhan, 1:800) were applied. Imaging J (v 1.8.0) was used to analysis the result.

### 2.5. Immunofluorescence (IF)

Paraffin sections were made using the tissue samples of the above samples. The paraffin sections were deparaffinized with water, sealed with hydrogen peroxide, then washed with double distilled water. Drop 0.01 mol/L, pH 7.4 PBS on the known antigen specimens, and discard them after 10 minutes to keep the specimens at a certain humidity. Add 0.01 mol/L, pH7.4 PBS appropriately diluted test antibody specimens to cover the known antigen specimens. Place the slides in an enamel box with a lid, and keep them warm at 37°C for 30 minutes. Take out the slides and place them on the slide rack, first rinse with 0.01 mol/L, pH 7.4 PBS for 1 to 2 times, and then sequentially immerse them in 0.01 mol/L, pH 7.4 PBS 3-cylinder, 5 minutes per cylinder, Oscillates from time to time. Take out the glass slide, use filter paper to absorb excess water, but without drying the specimen, add a drop of a certain dilution of fluorescently labeled anti-human globulin antibody. Place the slides flat in an enamel box with a lid, and keep them warm at 37°C for 30 minutes. Take out the glass slide, absorb the excess water with filter paper, add a drop of buffer glycerin, and cover it with a cover glass. Then, observe under a high-powered fluorescent microscope. Anti-VEGF polyclonal antibody (Servicebio, Wuhan, 1:800), anti-CD31 polyclonal antibody (Servicebio, Wuhan, 1:800) and anti- HIF-1α polyclonal antibody (Servicebio, Wuhan, 1:800) were applied.

### 2.6. Real-time fluorescence quantitative PCR (RT-qPCR) assay

Total RNA from skin tissues was extracted using TRIzol reagent according to standard protocols. Primers are listed in Table 2. RT-qPCR was performed in ABI QuantStudio 7 Flex Real-Time PCR system (Applied Biosystems, Foster City, CA). GAPDH was used as the internal housekeeping gene. Reaction procedure: 95°C pre-denaturation for 3 minutes, 95°C denaturation for 30 seconds, 60°C annealing extension for 30 seconds. This process included thirty circles. The relative level of target gene was calculated using the formula of 2^−ΔΔct^ (average ct of target gene − average ct of housekeeping gene).

**Table 2:** Primers and their sequences for RT-PCR analysis.

Primer	Sequence (5’–3’)
VEGF-hF	TCACCAAGGCCAGCACATAG
VEGF-hR	GAGGCTCCAGGGCATTAGAC
CD31-hF	TTCAACAGAGCCAACCACGC
CD31-hR	CTCCGATGATAACCACTGCAATAA
HIF-1α-hF	TTCCCGACTAGGCCCATTC
HIF-1α-hR	CAGGTATTCAAGGTCCCATTTCA

HIF-1α = hypoxia inducible factor-1α, VEGF = vascular endothelial growth factor.

### 2.7. Western blotting assay

Skin tissues were frozen in liquid nitrogen. Total protein was isolated in a lysis buffer, resolved by 10% sodium dodecyl sulfate polyacrylamide gel electrophoresis (SDS-PAGE) and transferred onto polyvinylidene fluoride membranes by electroblotting. Vascular endothelial growth factor (VEGF), CD31, and hypoxia inducible factor-1α (HIF-1α) proteins were detected using the anti-VEGF polyclonal antibody (1:1000 dilution, Servicebio, Wuhan), anti-CD31 polyclonal antibody (1:1000 dilution, Servicebio, Wuhan), anti- HIF-1α polyclonal antibody (1:1000 dilution, Servicebio, Wuhan). Add 1:5000 diluted secondary antibody and incubate at room temperature for 1 hour, and develop color by chemiluminescence method. The bands were visualized with an enhanced chemiluminescence kit (Millipore, Billerica, MA) and analyzed with Image-Pro Plus 6.0 (Media Cybernetics, US). The relative expression level of target protein was taken as the ratio of target protein to GAPDH band gray value.

### 2.8. Statistical analyses

The results are expressed as the mean ± SD. Statistical analyses were performed using SPSS 24.0 software. Statistical significance was determined by Student *t* test when 2 groups (RN group and N group) were compared. The radiated skin tissue was named RN group. The normal skin tissue was named N group. 12 samples from each group were used for experimental validation. Statistical significance was considered if the *P* value was <.05.

## 3. Results

### 3.1. Histological observation of the skin

**Figure 1.**
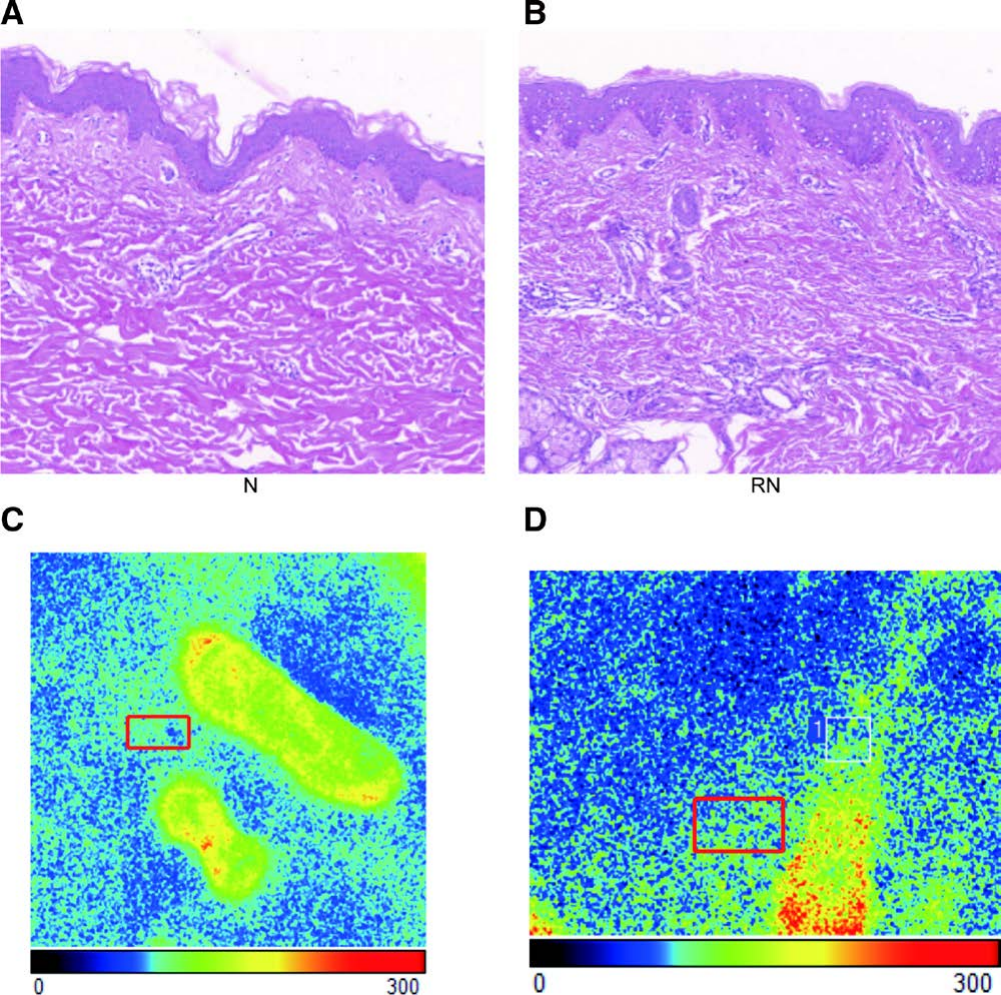
(A) Group N of normal tissue samples from patients with keloids. (B). Group RN of normal tissue samples from patients with recurrent keloids. (C) The average strength of group N by Doppler flow detector. (D) The average strength of group N by Doppler flow detector.

The cells were irregularly arranged, and badly damaged in keloid skin after radiotherapy, compared with the normal skin (Fig. 1A). There are more vacuoles in the epidermis (Fig. 1B). The local blood flow of group RN was enhanced. The blood perfusion of group N is 82.94 (Fig. 1C). The blood perfusion of group RN is 99.13 (Fig. 1D). The Radiotherapy treated skin tissue was still higher than that of Group N.

### 3.2. The expression level of VEGF, CD31, and HIF-1α via IHC

**Figure 2.**
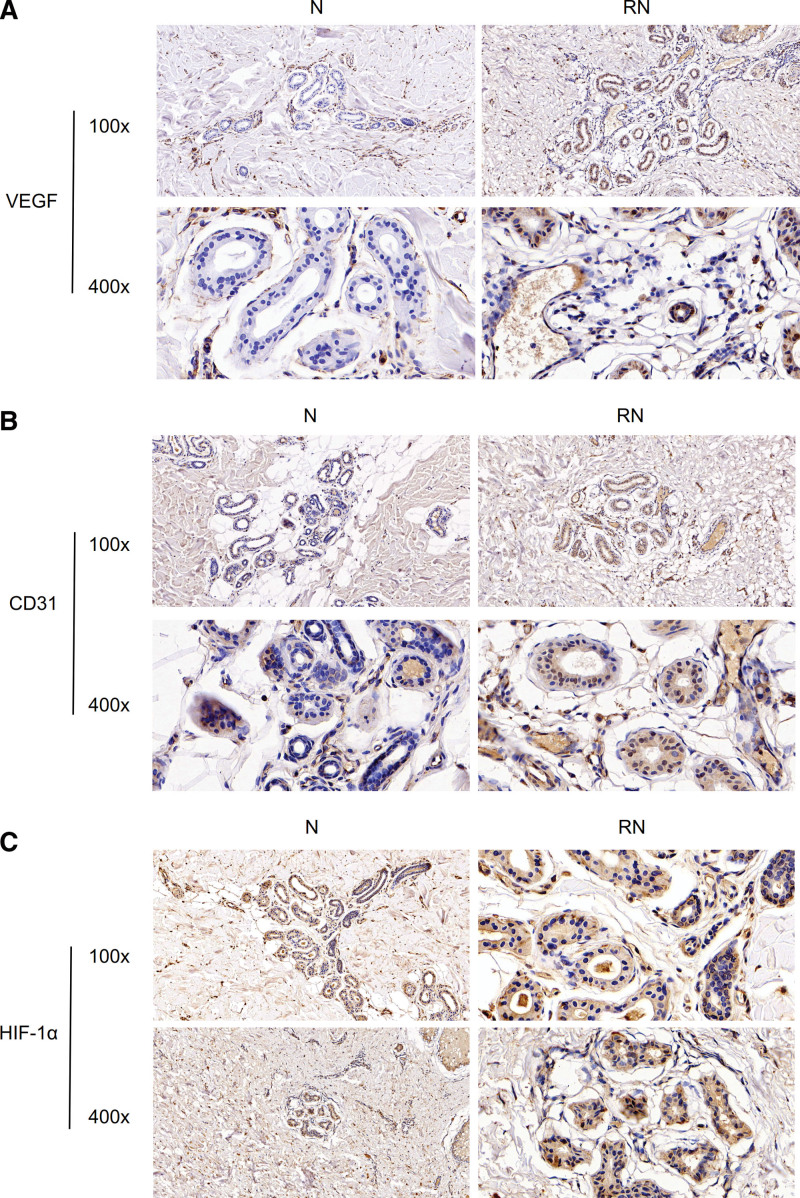
(A) IHC verification of VEGF between group N and RN. 100×. 400×.(B) IHC verification of CD31 between group N and RN. 100×. 400×. (D) IHC verification of HIF-1α between group N and RN. 100×. 400×. HIF-1α = hypoxia inducible factor-1α, IHC = immunohistochemistry, VEGF = vascular endothelial growth factor.

Via IHC, the expression of VEGF, CD31, and HIF-1α protein in the keloid-recurred skin (RN) group and the normal skin group were shown in Figure 2. The expression of VEGF was higher than the normal skin group (*P* < .05, Fig. 2A, Fig. S1A, http://links.lww.com/MD/H701). The expression of CD31 protein in the keloid-recurred skin (RN) group was higher than the normal skin group (Fig. 2B, *P* < .05, Fig. S1B, http://links.lww.com/MD/H701). The expression of HIF-1α protein in the keloid-recurred skin (RN) group was higher than the normal skin group (Fig. 2C, *P* < .05, Fig. S1C, http://links.lww.com/MD/H701).

### 3.3. The expression level of VEGF, CD31, and HIF-1α via IF

**Figure 3.**
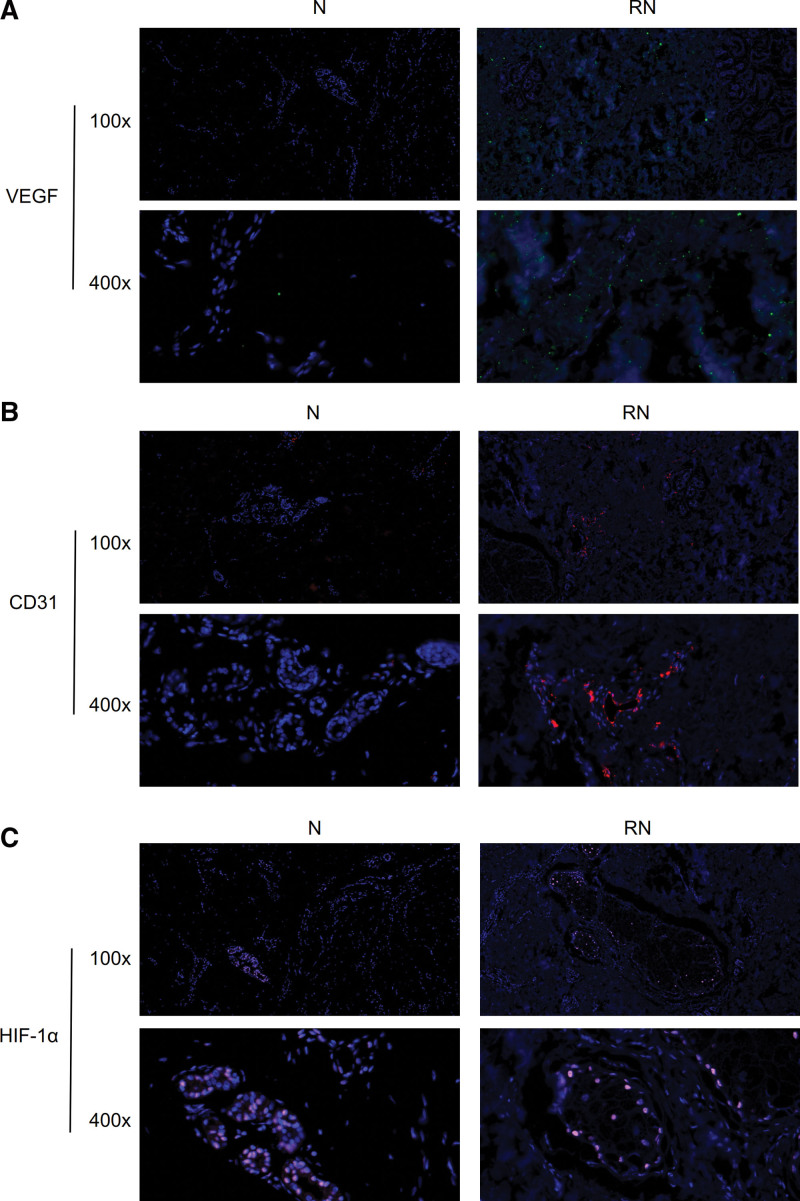
(A) Immunofluorescence verification of VEGF between group N and RN. 100×. 400×.(B) Immunofluorescence verification of CD31 between group N and RN. 100×. 400×. (D) Immunofluorescence verification of HIF-1α between group N and RN. 100×. 400×. HIF-1α = hypoxia inducible factor-1α, VEGF = vascular endothelial growth factor.

**Figure 4.**
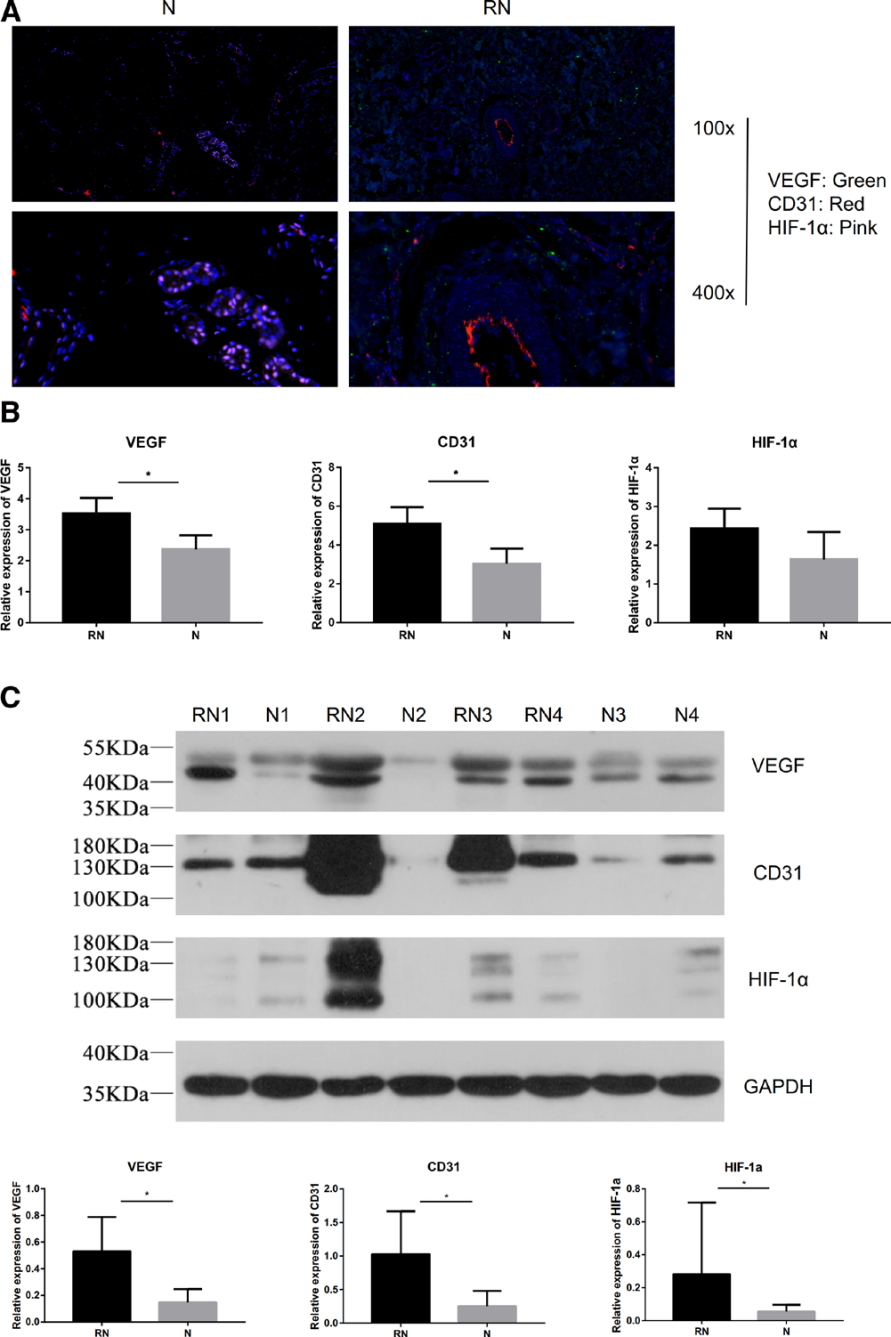
(A) Immunofluorescence verification of VEGF, CD31, and HIF-1α between group N and RN. (B) The relative expression level of VEGF, CD31, and HIF-1α between group N and RN by RT-PCR. (C) The relative expression level of VEGF, CD31, and HIF-1α between group N and RN by western blot. (D) The quantitative calculation of VEGF, CD31, and HIF-1α between group N and RN by western blot. HIF-1α = hypoxia inducible factor-1α, VEGF = vascular endothelial growth factor.

Compared with the normal skin group, the expression of VEGF and CD31 was up-regulated (Fig. 3A and B). However, there was no difference of HIF-1α expression between the RN and normal skin group through the IF (Fig. 3C). And the overview of these 3 proteins expression was manifested in the 3-channel fluorescence (Fig. 4A). The expression of VEGF was higher than the normal skin group (*P* < .01, Fig. S1D, http://links.lww.com/MD/H701). The expression of CD31 was higher than the normal skin group (*P* < .01, Fig. S1E, http://links.lww.com/MD/H701). The expression of HIF-1α tended to increase in RN group, but there was no significant difference between 2 groups (Fig. S1F, http://links.lww.com/MD/H701).

### 3.4. The comparison of expression via RT-qPCR

The results indicated that the relative expression level of VEGF and CD31 mRNA was significantly increased in RN group samples compared with those in the normal group (*P* < .05). However, there was no significant difference about the HIF-1α expression between the 2 groups (*P* > .05) (Fig. 4B).

### 3.5. The expression level of western blotting

Western blotting analysis showed that the expression of VEGF, CD31, and HIF-1α proteins were lower in the normal skin group than in the RN group (Fig. 4C). The expression of VEGF was higher in RN group than the normal skin group (*P* < .05). The expression of CD31 was higher in RN group than the normal skin group (*P* < .05). The expression of HIF-1α was higher in RN group than the normal skin group (*P* < .05).

## 4. Discussion

Keloid is a benign tumor of the skin mainly characterized by abnormal proliferation of fibrous tissue.^[4,11,12]^ Surgical resection of lesions with local electron beam therapy, that is, radiotherapy, is currently an effective treatment method. Shen^[5]^ had reported an effective rate of 88.25% with electronic beam therapy after keloid operation. Song^[13]^ even reported 94.03% efficacy rate combined surgical and radiotherapy with hyperbaric oxygen therapy. Even though, high recurrence rate still exists. Doornbos et al reported 23.5% recurrence rate in their study.^[14]^ With total doses between 10 and 20 Gy, Kutzner et al achieved a recurrence rate of 11.4%.^[15]^ Exploring effective method to decrease recurrence rate is still an important issue in keloid treatment. Analyzing the characteristics of recurred keloid and its surrounding tissue may offer some clues in exploring such methods. Microcirculation is pivotal factor influencing keloid development. We focused on skin microcirculation and endothelial cell function around recurred keloid in this study.

Blood perfusion has been deeply studied in keloid with noninvasive methods such as laser Doppler and LSCI. Timar-Banu et al compared the blood perfusion between different types of scars and normal skin with Laser Doppler flowmetry. They found that blood flow within keloids was higher than within normal skins.^[16]^ Liu et al measured blood perfusion of keloids, adjacent skin and nonadjacent skins with LSCI, their results showed that the perfusion within keloids and adjacent skin were significantly higher than nonadjacent skins.^[17]^ In this study, we measured the blood perfusion of radiated skin and normal skin in recurred keloid patients. The results indicates that the blood perfusion in radiated skin was higher than that of normal skin. This was the same with the result reported by Liu in which blood perfusion adjacent the keloid was higher than that of nonadjacent skin.^[17]^

VEGF is currently the widely studied pro-angiogenesis factors. The VEGF signaling pathway is involved in the entire process of angiogenesis. The proliferation of keloids is also closely related to VEGF-mediated endothelial cell proliferation and abnormal angiogenesis. Gira et al Compare the expression of messenger RNA between keloid and normal skin. High levels of VEGF expression have been observed in keloids.^[18]^ Fujiwara et al Compare the expression levels of VEGF and TGF-β1 in the dermis of keloid and normal skin. The results showed that in cultured keloid fibroblasts, the levels of mRNA and protein of these 2 markers were significantly higher than those of normal fibroblasts.^[19]^

Both in vivo and in vitro studies have proved that the angiogenic activity of keloid fibroblasts is higher than that of normal fibroblasts. In this study, the expression of VEGF in radiated skin in recurred keloid patients was higher than its surrounding normal skin. This suggested that radiotherapy after surgery did not curb the abnormal expression of VEGF induced by surgical trauma. the occurrence and development of keloids. Abnormal VEGF expression mediated endothelial cell proliferation and angiogenesis may be the potential factor of keloid relapse.

CD31 is a member of the immunoglobulin superfamily of adhesion molecules, which participates in the process of tumor cells adhering to endothelial cells and promoting tumor angiogenesis. CD31 may be used as an important indicator of a variety of tumor biological behaviors, and is closely related to tumor distant metastasis, clinical staging and prognosis.^[20]^ CD31 is closely related to the formation of blood vessels and is related to the movement of endothelial cells.^[21]^ When endothelial cells form new blood vessels, they first contact between cells, then proliferate, and then migrate to the surrounding matrix of blood vessels, where they reestablish cell connections and form new vascular cavities.^[22]^ Studies have shown that CD31 blocking antibodies can inhibit angiogenesis induced by cytokines and tumors in different animal models. The expression of CD31 in the mesothelioma cell line can induce blood vessel formation and promote cell movement.^[23]^ These experimental results indicate that during the formation of new blood vessels, CD31 may be involved in adhesion and signal transduction. Zhang et al calculated the microvessel density by CD31 staining, and there was a significant positive correlation between periosteal protein and the microvessel density observed by CD31 staining.^[24]^ Kurokawa et al used CD31 immunostaining to analyze vascularization, and used computers to construct 3-dimensional images of microvessels in keloids and hypertrophic scars.^[25]^ The same as VEGF, increased CD31 expression was also observed in radiated skin compared with the surrounding normal skin. Surgical trauma induced abnormal CD31 expression may also be the potential factor of keloid recurrence.

The proliferation of keloids is closely related to the proliferation of blood vessels in the microcirculation. HIF-1α is an important response factor under hypoxia. During the formation of keloids, the hypoxia microenvironment in local tissues can induce HIF-1α Express and regulate downstream angiogenesis, inflammation, apoptosis and other biological processes.^[26]^ Studies have shown that 2ME2 can effectively inhibit the protein expression of HIF-1α, and significantly increase the apoptosis of keloid fibroblasts induced by radiation.^[27]^ VEGF165 in the VEGF family is the member most closely related to the proliferation and angiogenesis of vascular endothelial cells. It exerts corresponding biological effects by binding to the receptors Flt-1 and Flk-1 on the endothelial cell membrane.^[28]^ In this study, there was no significant difference in the results of IF, IHC, and WB in the radiation skin of patients with recurrent keloids and the surrounding normal skin, but there was a tendency to increase in the RN group. This means that HIF-1α may also be a potential indicator for the treatment of keloids.

Although encourage results have been reached, there are still some shortcomings. First, we did not compare VEGF, CD31, and HIF-1α expression between recurred and no recurred keloid patient whose radiated skin sample could not be collected. Second, we did not conduct bioinformatics analysis to identify the mechanic role of VEGF, CD31, and HIF-1α in keloid development and recurrences. The most important, we did not analyze the relationship between radiation energy and the expression of VEGF, CD31, and HIF-1α. Although there are individual differences between patients. This may help to determine the most effective radiation method in keloid radiotherapy. We will try to carry out such study in the future.

## 5. Conclusions

There are significant differences in the expression of VEGF, CD31, and HIF-1α in radiated skin and its surrounding normal skin in recurred keloid patients. They may be used as potential biomarkers and targets for future research on keloid recurrence and treatment. The changes of blood vessels after keloid surgery and radiotherapy may be an important factor affecting the therapeutic effect. The changes of blood vessels in the skin of recurrent patients after radiotherapy can clarify this effect and even serve as the therapeutic effect and recurrence biomarker.

## Authors contribution

WL and MS did experiments and wrote draft. YH and HL carried out experimental technical support. JQ and YW designed the study and revised the manuscript.

**Conceptualization:** Mengjie Shan, Jie Qiu, Youbin Wang.

**Data curation:** Wenbo Li, Hao Liu.

**Formal analysis:** Yan Hao.

**Funding acquisition:** Mengjie Shan, Jie Qiu.

**Investigation:** Wenbo Li.

**Methodology:** Mengjie Shan.

**Project administration:** Mengjie Shan, Youbin Wang.

**Resources:** Mengjie Shan, Yan Hao, Youbin Wang.

**Software:** Yan Hao.

**Validation:** Jie Qiu.

**Writing – original draft:** Mengjie Shan, Yan Hao.

**Writing – review & editing:** Youbin Wang.

## Supplementary Material


