# Supplementary material for: Skin endothelial cell and microcirculation function study in recurred keloids patients after keloid surgery and radiotherapy
Source: Medicine (Baltimore). 2022 Oct 28;101(43):e31286. doi: 10.1097/MD.0000000000031286 (PMC9622619; doi:10.1097/MD.0000000000031286)

Supplementary figure 1. (A-C) The expression level of VEGF, CD31, and HIF-1α via immunohistochemistry-score. (D-F) The expression level of VEGF, CD31, and HIF-1α via immunofluorescence-score.

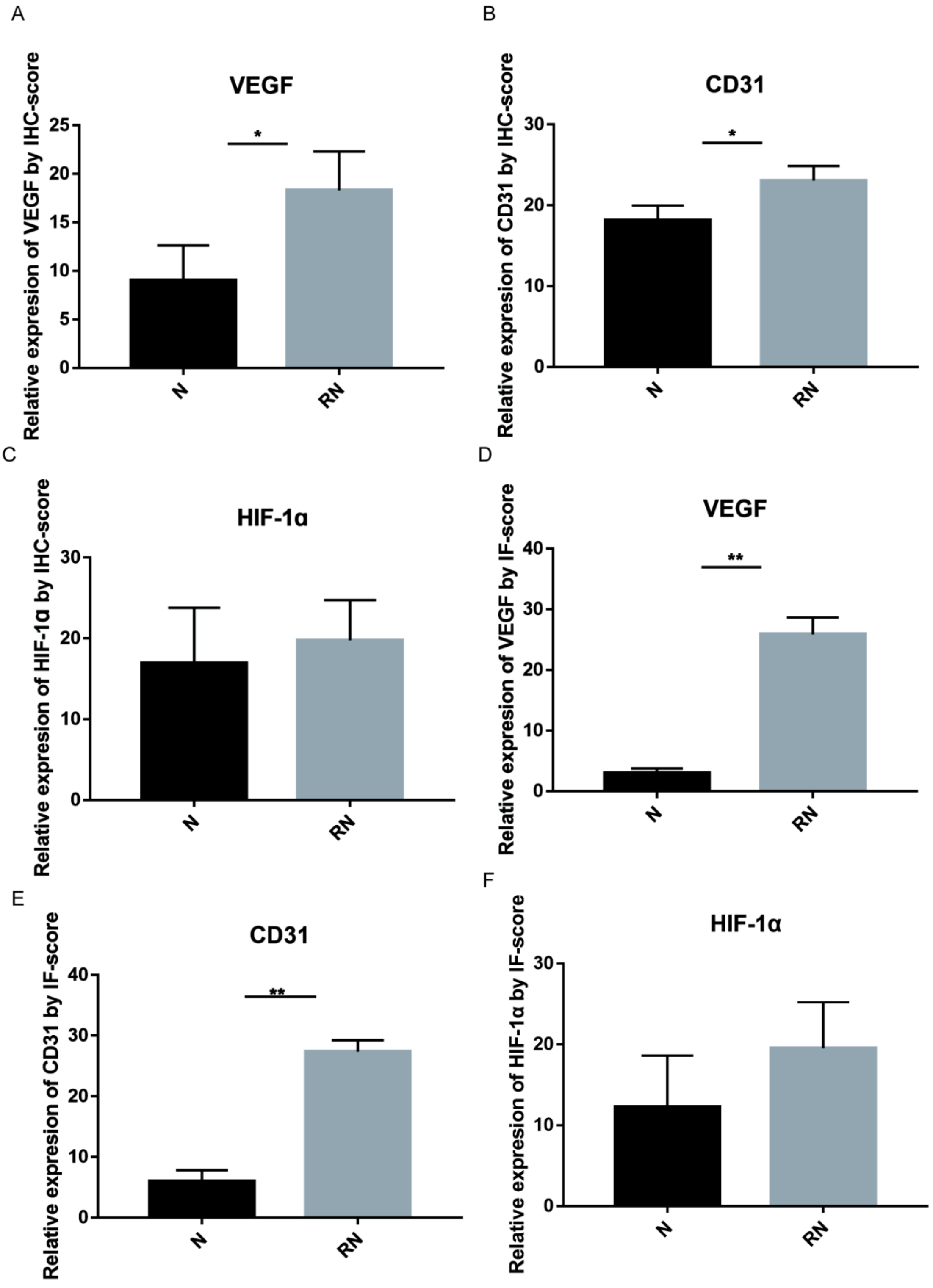

Supplement: Supplementary file 1 [file medi-101-e31286-s001.pdf]
